# Prediction model construction of mouse stem cell pluripotency using CpG and non-CpG DNA methylation markers

**DOI:** 10.1186/s12859-020-3448-3

**Published:** 2020-05-04

**Authors:** Soobok Joe, Hojung Nam

**Affiliations:** School of Electrical Engineering and Computer Science, Gwangju Institute of Science and Technology (GIST), Buk-gu, Gwangju, 61005 Republic of Korea

**Keywords:** DNA-methylation, Stem cell pluripotency, Non-CpG methylation

## Abstract

**Background:**

Genome-wide studies of DNA methylation across the epigenetic landscape provide insights into the heterogeneity of pluripotent embryonic stem cells (ESCs). Differentiating into embryonic somatic and germ cells, ESCs exhibit varying degrees of pluripotency, and epigenetic changes occurring in this process have emerged as important factors explaining stem cell pluripotency.

**Results:**

Here, using paired scBS-seq and scRNA-seq data of mice, we constructed a machine learning model that predicts degrees of pluripotency for mouse ESCs. Since the biological activities of non-CpG markers have yet to be clarified, we tested the predictive power of CpG and non-CpG markers, as well as a combination thereof, in the model. Through rigorous performance evaluation with both internal and external validation, we discovered that a model using both CpG and non-CpG markers predicted the pluripotency of ESCs with the highest prediction performance (0.956 AUC, external test). The prediction model consisted of 16 CpG and 33 non-CpG markers. The CpG and most of the non-CpG markers targeted depletions of methylation and were indicative of cell pluripotency, whereas only a few non-CpG markers reflected accumulations of methylation. Additionally, we confirmed that there exists the differing pluripotency between individual developmental stages, such as E3.5 and E6.5, as well as between induced mouse pluripotent stem cell (iPSC) and somatic cell.

**Conclusions:**

In this study, we investigated CpG and non-CpG methylation in relation to mouse stem cell pluripotency and developed a model thereon that successfully predicts the pluripotency of mouse ESCs.

## Background

DNA methylation is crucial in epigenetic control and is the best-studied epigenetic variation in mammals. DNA methylation is important in silencing retroviral elements, in regulating tissue-specific gene expression, in genomic imprinting, and in X chromosome inactivation. Research also suggests that epigenetic regulation is essential to maintaining the pluripotency of embryonic stem cells (ESCs) [1–5]. The process by which ESCs maintain their pluripotency is precisely controlled by cell-specific regulation, and several epigenetic factors, along with gene expression, appear to be involved therein: these molecular-level mechanisms are perceived as varying dynamically per cell. While there is a possibility that pluripotent ESCs can be used in various medical fields [6–8], their use in clinical practice requires a precise understanding and control of the functions undergirding ESC pluripotency [9].

Medical procedures, such as hematopoietic stem cell transplantation, have proven that stem cell research is important for regenerative medicine [10, 11]. Generally, stem cells are well known to have the potential to regenerate and repair damaged cells and tissues. A stem cell having higher pluripotency means a stem cell can divide into any type of cell. Thus, understanding the level of pluripotency is very essential in the stem cell study. In this perspective, a more accurate and specific model has become necessary to understand and predict cellular pluripotency. For predicting the pluripotent capacity of stem cells, two primary methods have been utilized, including PluriTest [12], which is based only on gene expression, and Epi-Pluri-Score [13], which is based on CpG DNA methylation. Using next-generation sequencing technology, however, researchers have also discovered DNA methylation in ESCs in areas other than CpG sequences, called non-CpG DNA methylation [14–17]. Non-CpG methylation sites, which are rarely found in normal adult cells, have primarily been observed in ESCs or brain tissues: Among adult cells, only 0.02% exhibit methylation at non-CpG sites. In stem cells, however, 10 to 25% of all cytosine methylations are reported in non-CpG regions [18, 19]. Accordingly, studies of stem cell pluripotency may benefit from implementing approaches that consider both CpG and non-CpG methylation. In support thereof, a recent study deemed that non-CpG methylation can be used as a biomarker for assessing endodermal pluripotency capacity [20]. However, only limited measures of DNA methylation were used to document non-CpG methylation by Illumina array, and the authors focused only on differences in overall amounts of methylation. Furthermore, although non-CpG methylation is relatively common in pluripotent stem cells, a clear understanding of the role of non-CpG methylation in ESCs across the stem cell pluripotency spectrum is lacking.

**Fig. 1:**
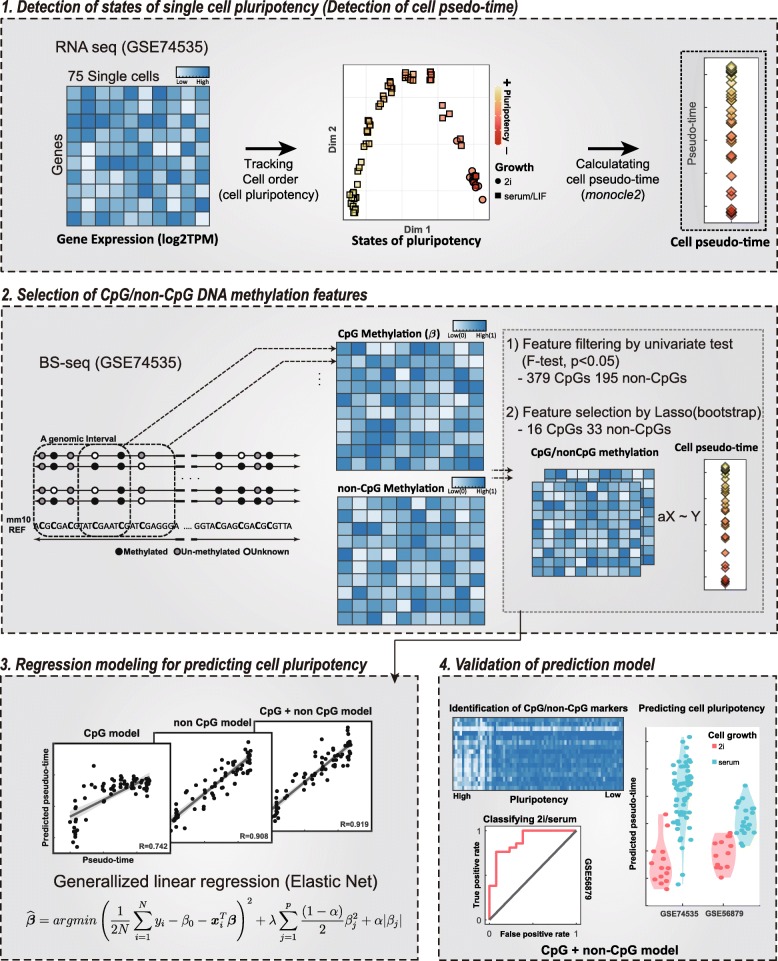
Overview of epigenetic and transcriptomic analysis of single stem cells for pluripotency prediction. In total, 75 mouse single embryonic stem cells (GSE74535), for which gene expression and DNA methylation were sequenced simultaneously, were used to generate a linear model that predicts cell pluripotency. The biological pseudo-time of each cell was determined using gene expression data. Using the CpG and non-CpG DNA methylation levels of each genomic interval, we constructed a linear model that predicts cell pseudo-time. Validation was performed using DNA methylation sequencing data from 34 independent samples of single and bulk ESCs (GSE56879)

Hypothesizing that non-CpG methylation may be an important biomarker of stem cell pluripotency, we developed a novel machine learning model for predicting mouse stem cell pluripotency based on CpG and non-CpG DNA methylation markers, and using the model, we sought to evaluate DNA methylation changes in relation to degrees of pluripotency in stem cells. To construct the machine learning model, we used gene expression and DNA methylation data obtained through parallel RNA and DNA sequencing of 75 mice single ESCs. To determine the degrees of pluripotency in individual ESCs, we relied on cell-specific pseudo-time estimated using gene expression data for single cells. Using this cell pseudo-time as a gold standard, we found that states of cell pluripotency for mouse single ESCs, cells in the development stage, and induced mouse pluripotent stem cells (iPSCs) could be predicted according to DNA-methylation levels at CpG and non-CpG sites (Fig. 1).

## Results

### Gene expression based on pseudo-time in single cells represents pluripotency well

**Fig. 2:**
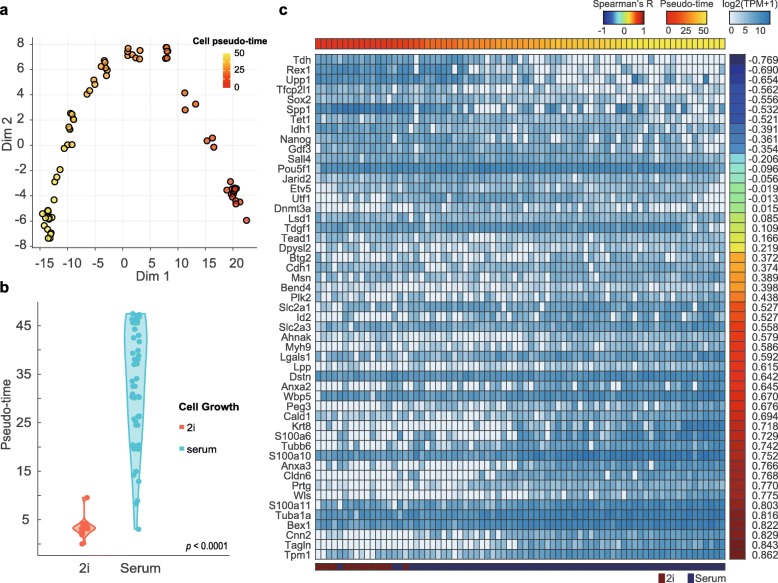
Pseudo-time of single ESCs based on gene expression. **a** Two-dimension plot of 75 single cells based on gene expression data, applying monocle2. Color bar represents cell pseudo-time based on gene expression data. **b** A violin plot of pseudo-times of cells according to cell culture environment. Red dots represent pseudo-time for cells grown in 2i media; blue dots represent cells cultured in serum. *p* represents a result of ranksum test between cells in 2i and serum environments by Wilcoxon rank-sum test. **c** A heat map of gene expression ordered using the monocle2 cell ordering method. Pluripotent/differentiation marker genes are sorted according to Spearman correlation coefficients between cell pseudo-time and gene expression levels. Samples are sorted by cell pseudo-time detected by the monocle2 method. Numbers in the right columns indicate Spearman’s correlation coefficients; top red-yellow color bar represents the cell pseudo-time; and the bottom blue/red color bar indicates the environment for cell growth

In order to construct a machine learning model with which to predict the pluripotency of a single cell, first, we needed to define the degree of pluripotency for every ESC in the dataset. The pluripotency of a single ESC is based on gene expression from RNA sequencing data, and pluripotency may differ across a set of cells. To construct a pseudo-time thereof for cell ordering, gene expression can be used to highlight sequential relationships among cells at different states of pluripotency. Here, we defined the pluripotency order of 75 single cells using three published cell-cell ordering methods based on gene expression, including monocle2 [21], SLICER [22], and TSCAN [23]. The basic assumptions in cell ordering are that cell pluripotency follows a defined pathway and that gene expression is correlated with the progression thereof. Next, we investigated correlations among the cell-cell ordering methods. The cell orders achieved with SLICER and TSCAN were similar to that of monocle2: Spearman’s correlation coefficients for cell order relationships were 0.93 between monocle2 and SLICER and 0.66 between monocle2 and TSCAN. All cell orders defined with the three methods were highly correlated with the expression patterns of known pluripotent marker genes, reflecting both cells in a ground state or near the completion of differentiation, as previously described in research on ESCs [24]. Throughout the rest of our study, we used the monocle2 method, as it had the highest correlation with conventional pluripotent genes among the three cell ordering methods (Supplementary Figure 1). According to cell-cell ordering, we estimated cell pseudo-time as the distance of gene expressions between individual cells using the monocle2 approach. We confirmed that cells collected at pseudo-time zero were clustered near cells grown in 2i media and that cells grown in a serum environment had higher pseudo-times (Fig. 2a). We also confirmed that the difference was significant between pseudo-times in cells in 2i and serum environments (Fig. 2b). The expression of genes previously identified as pluripotent markers was strongly correlated with cell pseudo-time. After determining cell pseudo-time, we noted that known stem cell markers were strongly enriched for a total of 4935 genes. Of 51 genes known to be conventional pluripotent markers, we confirmed that cell pseudo-time and the expression of most of the marker genes were highly correlated (based on Spearman’s correlation coefficients) when using monocle2 (Fig. 2c).

The expressions of *Rex1* (*R* = − 0.69), *Nanog* (*R* = − 0.36), *Tet1* (*R* = − 0.52), *Idh1* (*R* = − 0.39), and *Sox2* (*R* = − 0.56) tended to be negatively correlated with pseudo-time as defined in this study. Meanwhile, the expressions of *Bex1* (*R* = 0.82), *Cnn2* (*R* = 0.83), and *Tpm1* (*R* = 0.86) showed positive correlations with expression values according to biological pseudo-time (Fig. 2c, Supplementary Figure 2). These correlations confirmed that the degrees of pluripotency of stem cells were due to the expression of known marker genes and that the degrees of pluripotency of cells were well defined by pseudo-time based on gene expression profiles of stem cells.

### CpG and non-CpG methylation decreases with cell pluripotency

**Fig. 3:**
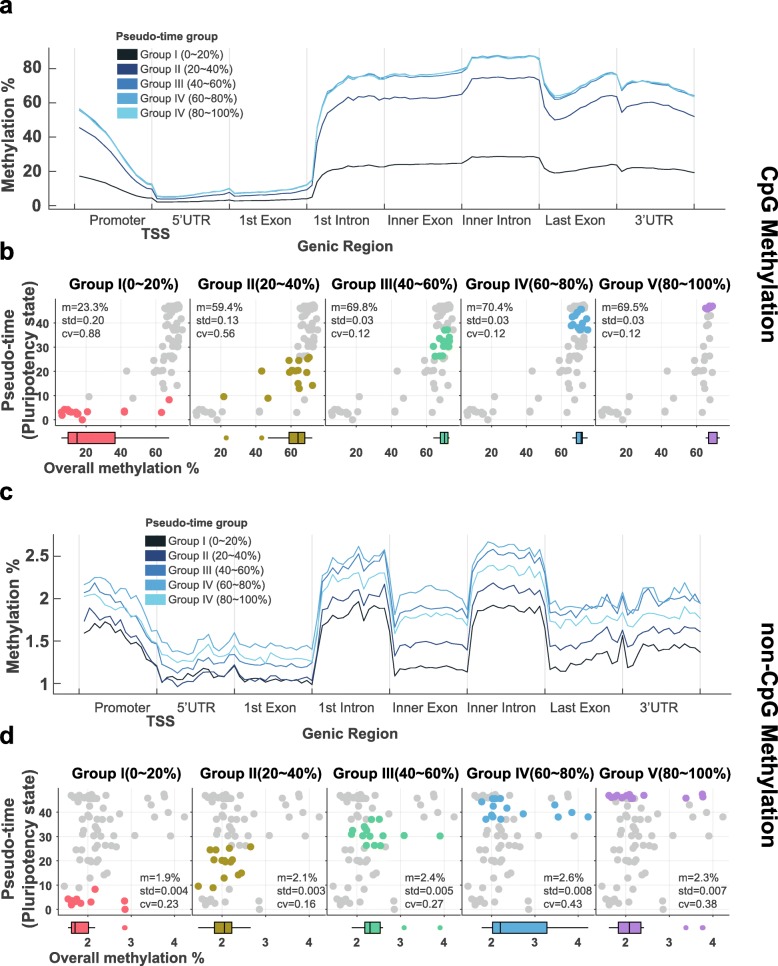
Overall DNA methylation levels according to cell pseudo-time. **a** Levels of overall CpG methylation and CpG methylation relative to gene structure. **b** For each group, the relation between overall CpG methylation and stem cell pseudo-time is provided. **c** Levels of overall non-CpG methylation and non-CpG methylation relative to gene structure. **d** For each group, the relation between overall non-CpG methylation and stem cell pseudo-time is provided. For (**a**,**c**), genic regions were split into 120 non-overlapping windows to determine methylation levels. Each region was divided into 12 small windows for methylation analysis. Percent values represent the pseudo-times of the cells: groups I to V represent the degrees of cell pluripotency, with group I having the shortest pseudo-time (pluripotency-high) and group V the longest (pluripotency-low). For (b,d), the m and cv values represent mean methylation levels and coefficients of variation, respectively

Moving forward, we investigated the global characteristics of DNA methylation in individual single cells across different states of pluripotency. More specifically, we examined DNA methylation across the region 1500 bp upstream from the transcription start site, the region 1500 kb downstream from the transcription end site, and the gene body region. To describe distributions of DNA methylation in greater detail, we assessed DNA methylation at CpG and non-CpG sites and investigated the methylation of CpG and non-CpG sites near the transcription start sites (TSSs) for all genes. For all single cells, methylation levels near TSSs, which represent promoter regions for first exons, were lower than methylation levels at other genomic regions (Fig. 3a,b). This is in keeping with a previous study that reported that about 65% of genes have CpG islands in their promoter regions and that most of these CpG islands remain un-methylated [25]. Moving away from first exons, both CpG and non-CpG methylation levels gradually increased in the first intron region. Interestingly, the non-CpG methylation levels of exons and 3’UTRs were less than those of introns. Comparing DNA methylation and cell pluripotency, we discovered that CpG DNA methylation across the entire genome gradually increased as cell pseudo-time increased (Fig. 3a,b). Similar to CpG DNA methylation, non-CpG regions tended towards slight increases (Fig. 3c,d). These results indicated negative correlations between degrees of pluripotency for ESCs and both overall CpG DNA methylation and overall non-CpG DNA methylation. Similar results in regards to methylation status and pluripotency were obtained for cells grown in different culture media. Evaluating methylation at CpG and non-CpG sites, we confirmed that cells cultured in 2i were more pluripotent and had significantly lower methylation levels (*P* value < 0.05, *t*-test) than cells cultured in serum. Indeed, the CpG and non-CpG methylation levels in cells (from GSE74535 and GSE56879) cultured in 2i were lower than those in cells grown in serum (see also Supplementary Figure 3a-b). Accordingly, we deemed that the total amounts of methylation in individual ESCs increase at both CpG and non-CpG regions with greater cell pseudo-time values. To compare increases in CpG and non-CpG methylation, we divided 75 samples into five groups according to their cell pseudo-times: using pseudo-time order, we divided the cells into equal numbers from group I to group V. Interestingly, overall CpG methylation levels remained stable from the third group with almost no increase thereafter (Fig. 3b). As for non-CpG methylation, however, while overall increases were noted, we noted that methylation levels were dispersed more evenly across pseudo-times (Fig. 3d). Accordingly, we deemed that changes in CpG methylation are more stable than those in non-CpG methylation and that methylation trends in non-CpG markers are similar to those in CpG markers, although with greater variability in pseudo-times.

### CpG and non-CpG methylation markers accurately predict ESC pluripotency

**Fig. 4:**
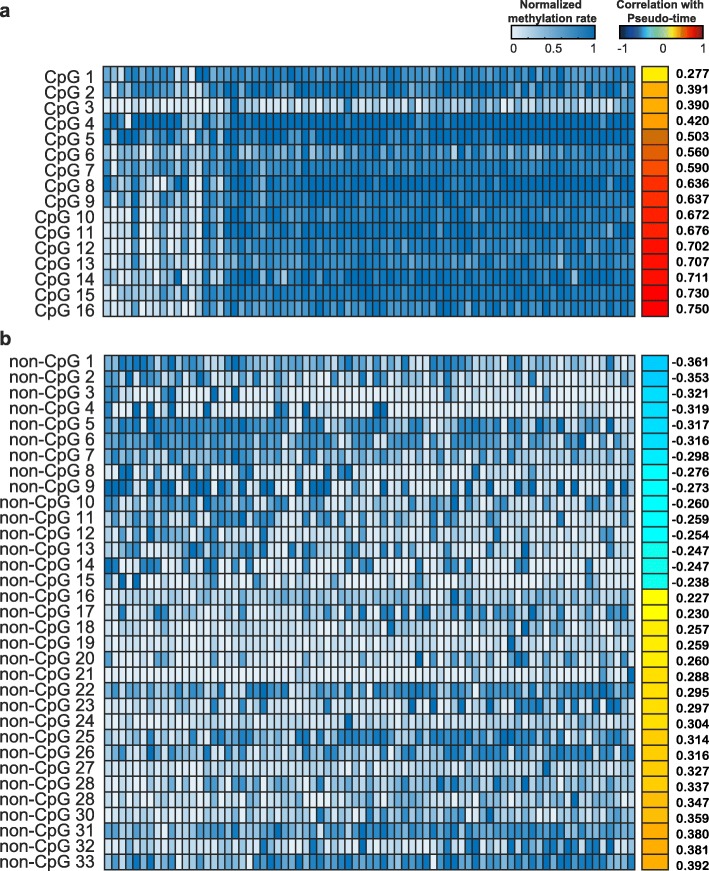
CpG and non-CpG markers for predicting stem cell pluripotency. **a** A heat map of methylation levels for 16 CpG markers. Samples are sorted by cell pseudo-times detected by the monocle2 method. **b** A heat map of methylation levels for 33 non-CpG markers. Samples are sorted by cell pseudo-time detected by the monocle2 method. The CpG and non-CpG markers are sorted according to Spearman correlation coefficients between cell pseudo-time and methylation levels for each marker. Numbers in the right column represent Spearman’s correlation coefficients

**Fig. 5:**
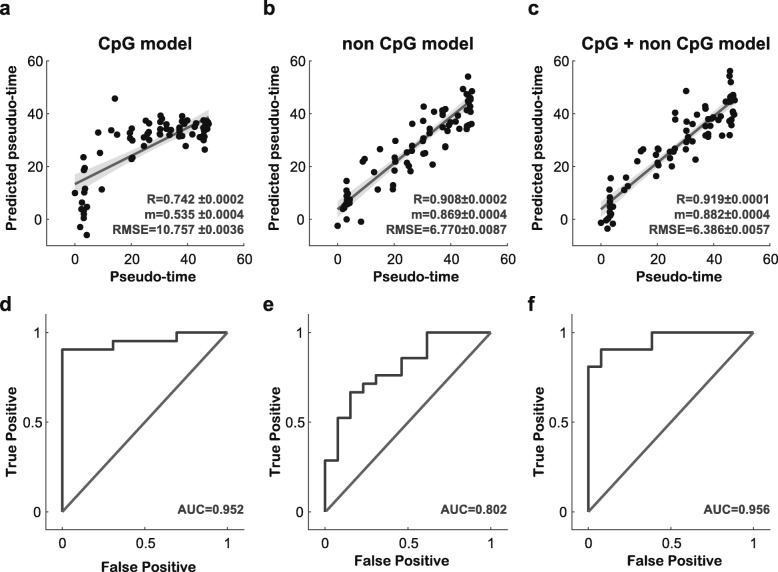
Prediction of cell pseudo-time via CpG and non-CpG methylation levels. (**a**-**c**). Correlation scatter plot of comparisons between cell pseudo-time and predicted cell time. In (**a**) and (**b**), each model used features that included a single cytosine type, CpG or non-CpG, respectively. In **c**, the model used a combination of markers for CpG and non-CpG sites. The fitted red lines represent a least-squares line for each scatter plot. R represents Pearson’s correlation coefficients, and m indicates the slopes of fitted linear lines. RMSE represents the root mean squared error between cell pseudo-time and predicted pseudo-time. (**d**-**f**) Receiver operating characteristic curves for classification of cells grown in 2i media or serum for the CpG, non-CpG, and combined models, respectively

To construct a model for predicting cell pluripotency in ESCs, we identified methylation markers for both CpG and non-CpG genomic intervals using the elastic net approach. We constructed three elastic net-based linear regression models (CpG, non-CpG, and combined models) using DNA methylation levels as features and cell pseudo-time as the gold standard. The numbers of DNA methylation markers were 16 and 33 for the CpG and non-CpG models, respectively. The combined model was based on the methylation of all cytosine residues regardless of cytosine type, comprising 49 marker intervals (16 CpG markers, 33 non-CpG markers). Interestingly, all CpG markers were positively associated with degrees of cell pseudo-time (Fig. 4a, Supplementary Table 1). While both positive and negative correlations were observed for non-CpG markers, most of them exhibited positive correlations with cell pseudo-time, similar to CpG markers (Fig. 4b, Supplementary Table 1). Next, to validate the performance of our model, leave-one-out cross-validation was initially conducted with 75 samples in the training set. We confirmed good performance for all models, including the combined model, in the internal test (Fig. 5a-c). Overall, the combined model showed the best performance in predicting cell pseudo-time and cell culture environment: Pearson’s correlation coefficient of 0.919, root-mean-square error of 6.386, and slope of 0.882 (Fig. 5c). For verification in an independent dataset, we used 34 instances of single-cell (32 instances) and bulk-cell BS-seq (two instances) data for cells cultured in serum and 2i media. Since gene expression values in the independent set were not provided, we sought to determine how these two cell groups could be distinguished in our model. A receiver operating characteristic curve was drawn based on estimated cell pseudo-time values derived from the model for the two groups (Fig. 5d-f). The area under the curve (AUC) of the combined model was 0.956 (Fig. 5f), and its accuracy was 91.2% (Supplementary Figure 4). Although we confirmed the classification performance of the 2i and serum groups due to the absence of gene expression, we clearly saw a difference in the predicted pseudo-times of each group (Supplementary Figure 5). In addition, the classification performance of the combined model showed similar or better performance than the CpG and non-CpG models (Supplementary Figure 4). These results indicated that cell pluripotency could be predicted based on CpG and non-CpG DNA methylation markers. Applying the combined model with the elastic net method and estimated cell pseudo-time, we noted that estimated cell pseudo-times were low in single cells grown in 2i (Supplementary Figure 5). Accordingly, the results of our predicted model based on CpG and non-CpG methylation markers showed that more single cells cultured in 2i were in a ground state than cells grown in serum.

### Prediction of pluripotency of iPSCs and ESCs according to developmental stage

To confirm that the cell pluripotency of other types of cells could be predicted by using our models, especially iPSCs, we used a public dataset and identified pseudo-times of different cell types. To do so, we collected BS-seq data and measured the methylation levels in the same manner as that in our previous experiments: the two public datasets had GEO numbers GSE64115 (induced pluripotent stem cells [iPSCs] and somatic cells) and GSE84235 (developmental stage of ESCs). To verify the performance of the pluripotency prediction model, we investigated pseudo-times for iPSCs and somatic cells according to developmental stage. Unfortunately, no dataset covered all of the methylation markers extracted from the training set; therefore, we proceeded to select common markers and to apply a model built only from these markers. The numbers in third column in Table 1 indicate the number of used markers. The results of our pluripotency prediction model revealed that the samples of the early cell development stage (E3.5) had lower cell pseudo-times than E6.5 samples (Table 1). Furthermore, we confirmed that the pseudo-time values of iPSCs were indeed less than those of somatic cells. This showed that our prediction markers of cell pluripotency could help with identifying cellular development stage and with determining the pluripotency of iPSCs.

**Table 1:** Prediction of cell pluripotency of ESCs and iPSCs. Prediction of pseudo-times of iPSCs, somatic cells, and stem cells according to development stage using the proposed pluripotency prediction model

Dataset	Samples	Number of markers used	Pseudo-time
GSE64115	WT MEF	10	24.30
GSE64115	WT iPSC	10	15.61
GSE84235	E6.5 (Proximal Epiblast)	48	15.35
GSE84235	E6.5 (Proximal Epiblast)	48	15.60
GSE84235	E6.5 (Extraembryonic Ectoderm)	48	13.88
GSE84235	E6.5 (Extraembryonic Ectoderm)	48	14.49
GSE84235	E3.5 (Inner Cell Mass)	48	5.36
GSE84235	E3.5 (Inner Cell Mass)	48	5.4
GSE84235	E3.5 (Trophectoderm)	48	5.24
GSE84235	E3.5 (Trophectoderm)	48	5.68

## Discussion

In this study, we noted several important characteristics of DNA methylation patterns in ESCs. First, the degrees of overall DNA methylation of ESCs changed dynamically (Fig. 3a-d). The number of cells with high cellular pluripotency showed low overall methylation levels for most CpG markers. This reflects the reported relationship between cell pluripotency and methylation described in previous studies [19, 26]. Meanwhile, non-CpG markers showed only small decreases in methylation with greater cell pluripotency. Second, our model based on CpG and non-CpG methylation performed well in predicting cell pluripotency. The cell pluripotency prediction model using different types of DNA methylation was constructed using the elastic net approach. The performance of the CpG and non-CpG combined model achieved a Pearson’s correlation coefficient of 0.919 when compared with cell pseudo-time based on transcriptomic pluripotency. Using an external dataset, we applied our models as classifiers to distinguish between 2i and serum environments. The combined model exhibited an AUC value of 0.956; the CpG and non-CpG models had AUC values of 0.952 and 0.802, respectively. The reason why both CpG and non-CpG markers could be used in a prediction model was that methylation levels for both were correlated with cell pluripotency, and these results suggested that non-CpG methylation could be a good marker for estimating cellular pluripotency. In addition, we investigated whether the prediction model could determine the degree of pluripotency in developing cells, as well as the degree of pluripotency in iPSCs and somatic cells. Our prediction model indicated a pattern of decreasing pluripotency as the cell develops and that predicted pseudo-times for iPSCs and somatic cells clearly differed. Thus, we deemed that our prediction model of the pluripotency of cells could be of use in stem cell research and pluripotency measurements of iPSCs (Table 1). As a limitation to our study, gene expression and DNA methylation information was obtained from single mouse ESCs, and we examined whole-genome regions despite low coverage and despite lacking detailed methylation information. We suspect that isolating more considerable multi-omics data including transcriptome and methylome data for single cells and including other mammalians will help with obtaining a more accurate representation of the role of DNA-specific methylation in ESC or iPSC pluripotency.

## Conclusions

To develop a prediction model of cell pluripotency, we investigated relationships between DNA methylation and pluripotency in single mouse ESCs and assessed the contributions of CpG and non-CpG-specific methylation to pluripotency. In doing so, we observed that DNA methylation differed with cell pluripotency and that epigenetic markers could be used to predict states thereof. We suggest that our prediction model of pluripotency based on both CpG and non-CpG DNA methylation markers successfully indicates the pluripotency of mouse ESCs.

## Methods

### Preprocessing and cell pseudo-time

scRNA-seq and scBS-seq parallel profiling data from mouse ESCs were obtained from a previous study with the Gene Expression Omnibus (GEO) accession ID GSE74535 [27]. Additional independent scBS-seq data were obtained with the GEO accession ID GSE56879 [28]. The GSE74535 dataset consisted of RNA and BS-seq data for 75 single cells (14 cells in a 2i environment, 61 cells in a serum environment); the GSE56879 dataset comprised only BS-seq data for 32 single and two bulk cells (13 cells in a 2i environment, 21 cells in a serum environment) from mice. As in the previous study [27], we also excluded samples in the GSE74535 dataset with a bisulfite-conversion efficiency of < 95% as estimated by non-CpG methylation. We conducted realignment processing from raw FASTQ files through a consistent process. All single cell datasets used in this study are available via the NCBI database under the Sequence Read Archive accession numbers SRP065548, SRP058091, and SRP041257. To detect expression at the gene level, alignment was performed using STAR software [29] with default parameters. Transcripts Per Million (TPM) values were also derived using the RSEM software package [30]. Before gene expression analysis, RNA-seq data were preprocessed. During this process, each gene with a TPM value greater than 1 in more than half of the samples was used. Finally, 4935 gene expression values from 75 samples were used in this study. For DNA methylation data, the first six base pairs were clipped off the 5′ end of raw sequence reads to remove the 6 N random priming portion in order to remove both poor-quality calls and adapters using Trim Galore! (www.bioinformatics.babraham.ac.uk/projects/trim_galore). The remaining sequences were then aligned to the mouse genome (build GRCm38) with Bismark [31] in the single-end mode (parameters: --non-directional). Duplicate sequences were excluded, and methylation calls were extracted. The cell ordering processes were conducted using three methods, monocle2 [21], SLICER [22], and TSCAN [23], applying their default parameter values. Cell pseudo-time was estimated by the monocle2 approach using all 4935 gene expression values of log2(TPM + 1).

### Identification of methylation levels

Methylation values for individual genomic loci were measured using a sliding window approach to increase the degree of genomic coverage and to overcome sparse BS-seq data. We measured DNA methylation levels by distinguishing cytosine from CpG sites and non-CpG sites. When the window size was *w* and the step size was *s*, for each genomic interval *l*, the methylation level of each interval *l* was identified as the mean methylation level of each binary single-base-pair cytosine methylation rate at an interval of *l*. The methylation level of each cytosine was defined as the ratio of methylated read counts and the sum of unmethylated and methylated read counts. If any sample was not found to have at least four covered cytosine bases in each genomic interval, those genomic intervals were discarded. Finally, for CpG DNA methylation, we constructed a CpG methylation matrix of 420 genomic intervals for the 109 mouse samples from the GSE74535 and GSE56879 datasets, with a window size of 3000 bp and a step size of 1500 bp (Supplementary Figure 6). For non-CpG methylation, we constructed a non-CpG methylation matrix of 3554 genomic intervals for the 109 mouse samples for both of CHH and CHG methylation data (in which H = A, T or C) from the GSE74535 and GSE56879 datasets.

### Linear regression modeling and statistical analysis

Prior to performing regression analysis, we performed a filtering task to select markers related to cell pluripotency in a large number of genomic intervals using the *f*-test. In this process, we used the mean methylation level of each genomic locus as an experimental variable and the pseudo-time of each single cell as a response variable. We then calculated *F*-statistic as the ratio regression sum of squares and mean square error through the univariate linear relationship between methylation level and pseudo-time. After the *f*-test, genomic intervals with *P* values less than 0.05 were selected. Filtering each bin group through the *f*-test with pseudo-time, intervals were reduced to 379 and 195 among CpG and non-CpG intervals, respectively. We then utilized the lasso [32] and elastic net [33] methods to select significant genomic intervals, and constructed predictive models for CpG, non-CpG, and a combination thereof. We first performed the bootstrap procedure 1000 times for lasso regression. After selecting CpG and non-CpG intervals, we defined more than half of the selected genomic intervals as the final epigenetic markers in 500 runs. Next, we applied the elastic net method, which is widely used to process high-dimensional variables with a small number of samples, for selecting prediction markers. In formulas (1) and (2) below, *y* represents the cell pseudo-time vector; *β*_*i*_ represents the coefficient of the *i* th genomic interval; and *x*_*i*_ represents the degree of methylation of the *i* th genomic interval. The elastic net approach uses the L1 and L2 normalization techniques, which are core concepts in lasso [32] and ridge [34] regression methods. Below, *α* is the penalty weight. When *α* is 0, it is identical to ridge regression, and when it is increased to 1, it more closely resembles lasso regression.

1 $$ \hat{\beta}=\arg \operatorname{}\underset{\beta_0,\beta }{\min}\left(\frac{1}{2N}\sum \limits_{i=1}^N{\left({y}_i-{\beta}_0-{x}_i^T\beta \right)}^2+\lambda {P}_{\alpha}\left(\beta \right)\right), $$

2 $$ {P}_{\alpha}\left(\beta \right)=\sum \limits_{j=1}^p\left(\frac{\left(1-\alpha \right)}{2}\right){\beta}_j^2+\alpha \left|{\beta}_j\right|. $$

All statistical tests and analyses were conducted using MATALB2018b and R3.5.2. For pseudo-time comparison, we conducted Wilcoxon rank sum test [35].

### Parameter selection for the elastic net approach

Among 420 CpG and 3554 non-CpG methylation genomic intervals defined using the raw bisulfite sequencing data, we selected only 49 genomic intervals through use of *f*-test and lasso regression. Next, the intervals of the prediction model were selected by the elastic net method. For linear regression models, we selected α and λ regularization parameters by a cross validation approach. We found α and λ values according to minimized root-mean square errors. As stated above, when α is zero, it is identical to ridge regression, and when α is 1, it is identical to lasso. When λ increases, the coefficients are shrunk more. For optimal λ values, 10-fold cross validation was performed using GSE74535 to select final parameters, and external validation was performed with GSE56879 data. When we treated the alpha values in similar ways, there were no differences when we adjusted the alpha; therefore, we treated alpha values as 1. This means the model used lasso regression and was simpler than ridge regression. Finally, all of prediction models were conducted with an optimal α of 1 and λ values (Supplementary Fig. 7).

### Induced pluripotent stem cells and ESCs according to developmental stage

For validation of model performance, two public datasets were used (GEO numbers GSE64115 and GSE84235). Again, methylation levels were investigated using the sliding window approach. To verify the additional performance of the model, we evaluated pseudo-times for iPSCs and somatic cells by using detected common methylation markers, and we also evaluated pseudo-times according to developmental stage based on public methylation data.

## Supplementary information

**Additional file 1: Figure S1.** Distributions of correlation coefficients between pluripotent and differentiation marker gene expressions and cell orders of each ordering method. **Figure S2**. Pluripotent gene expression levels according to cell pseudo-time. **Figure S3**. Overall CpG methylation and non-CpG methylation levels relative to cell culture environment. **Figure S4**. Prediction of cell culture environmnet by proposed model using external dataset. **Figure S5**. Distributions of estimated cell pseudo-times by linear regression analysis. **Figure S6**. A sliding window approach to define methylation levels at each genomic interval. **Figure S7**. Selection of λ values of pluripotency prediction models.

**Additional file 2: Table S1**. List of the 16 CpG and 33 non-CpG genomic ranges used in the combined prediction model. Each of the chr, start, and end columns indicate chromosome and location information. R is the Pearson’s correlation coefficient, and p is the f-test result *p*-value. The type column indicates a CpG or non-CpG region.

## Data Availability

We used public datasets for this study. Gene expression and methylation data that support the findings of this study have been deposited in Gene Expression Omnibus with the accession codes GSE74535, GSE56879, GSE64115, and GSE84235.

## Abbreviations

AUC: Area under the curve
ESC: Embryonic stem cell
GEO: Gene Expression Omnibus
iPSC: Induced pluripotent stem cell
TPM: Transcripts Per Million
TSS: Transcription start site
