# Supplementary material for: Prediction model construction of mouse stem cell pluripotency using CpG and non-CpG DNA methylation markers
Source: BMC Bioinformatics. 2020 May 4;21:175. doi: 10.1186/s12859-020-3448-3 (PMC7199378; doi:10.1186/s12859-020-3448-3)
Supplement: Supplementary file 1 — Additional file 1: Figure S1. Distributions of correlation coefficients between pluripotent and differentiation marker gene expressions and cell orders of each ordering method. Figure S2. Pluripotent gene expression levels according to cell pseudo-time. Figure S3. Overall CpG methylation and non-CpG methylation levels relative to cell culture environment. Figure S4. Prediction of cell culture environmnet by proposed model using external dataset. Figure S5. Distributions of estimated cell pseudo-times by linear regression analysis. Figure S6. A sliding window approach to define methylation levels at each genomic interval. Figure S7. Selection of λ values of pluripotency prediction models. [file 12859_2020_3448_MOESM1_ESM.docx]

# Additional files

## Additional File 1. Supplementary Figures

### Supplementary Figure 1. Distributions of correlation coefficients between pluripotent and differentiation marker gene expressions and cell orders of each ordering method.


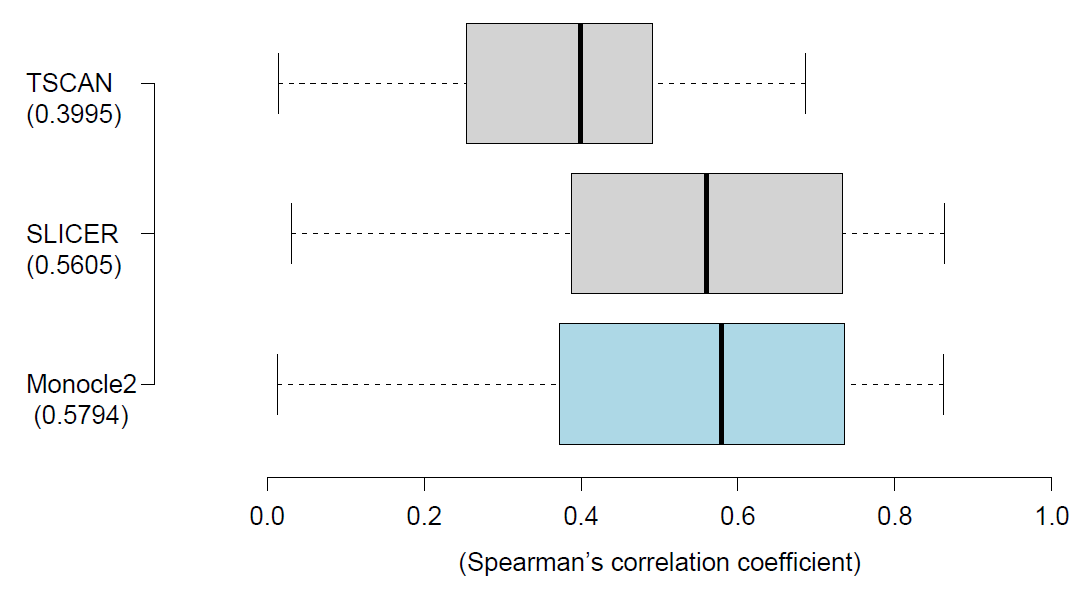


Boxplots depict absolute correlations between cell orders for each method and expression of known marker genes. Numbers in parentheses denote the median values of correlations for each method.

### Supplementary Figure 2. Pluripotent gene expression levels according to cell pseudo-time.


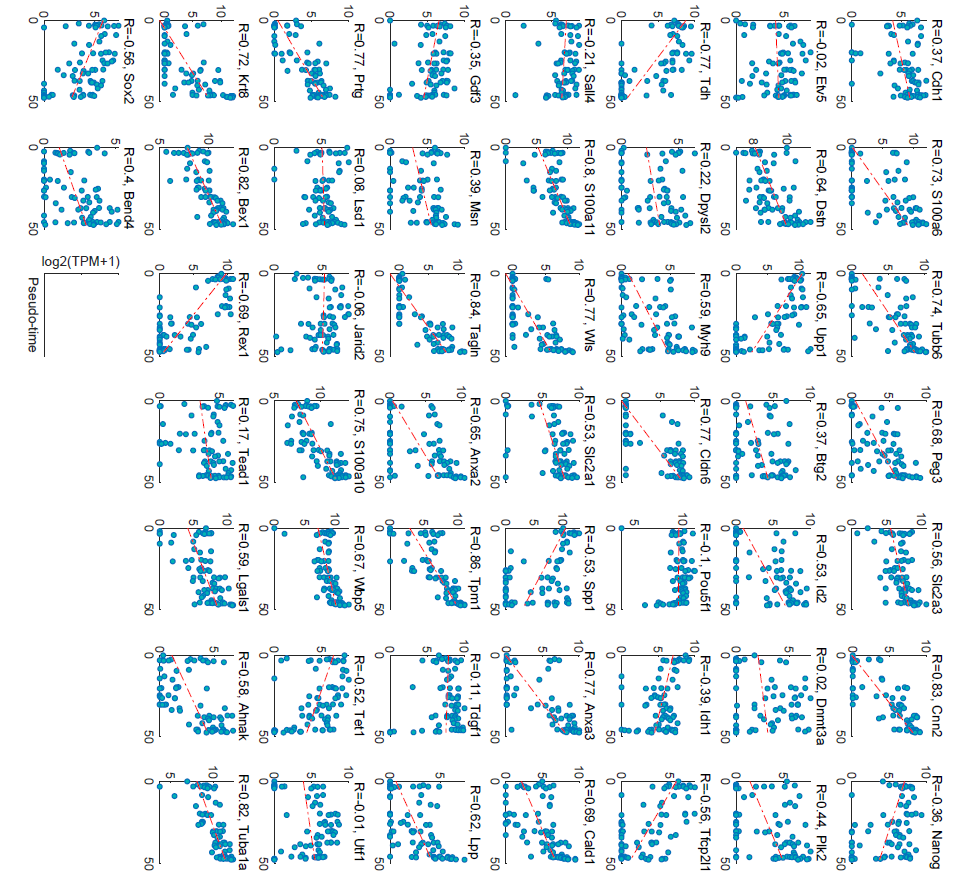


Expression levels of pluripotent or differentiation markers for a total of 51 genes are plotted for each cell pseudo-time. Red lines represent a least-squares line on each scatter plot.

### Supplementary Figure 3. Overall CpG methylation and non-CpG methylation levels relative to cell culture environment.


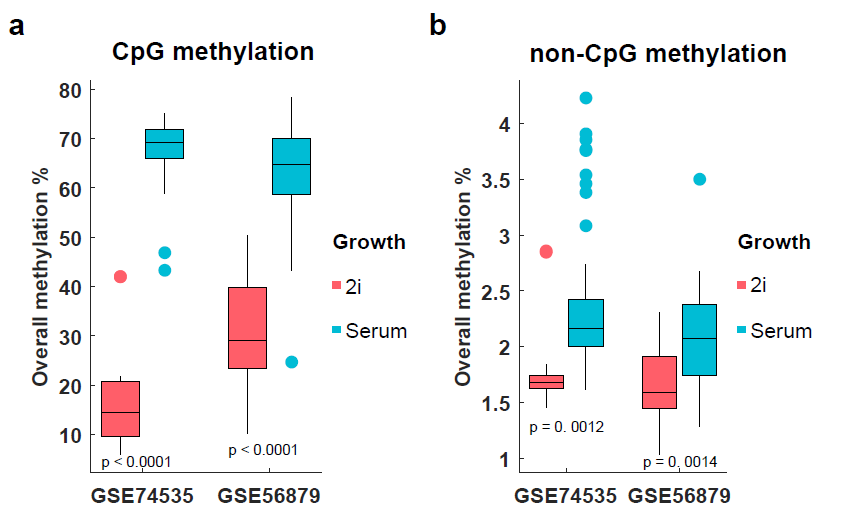


(a) Boxplot of overall CpG methylation levels according to cell culture environment. (b) Boxplot of overall non-CpG methylation levels according to cell culture environment. Red boxes represent pseudo-times for cells grown in 2i media, and blue boxes represent cells cultured in serum. *p* represents the significance of differences in overall methylation levels between 2i and serum culture groups according to t-test.

### Supplementary Figure 4. Prediction of cell culture environmnet by proposed model using external dataset.


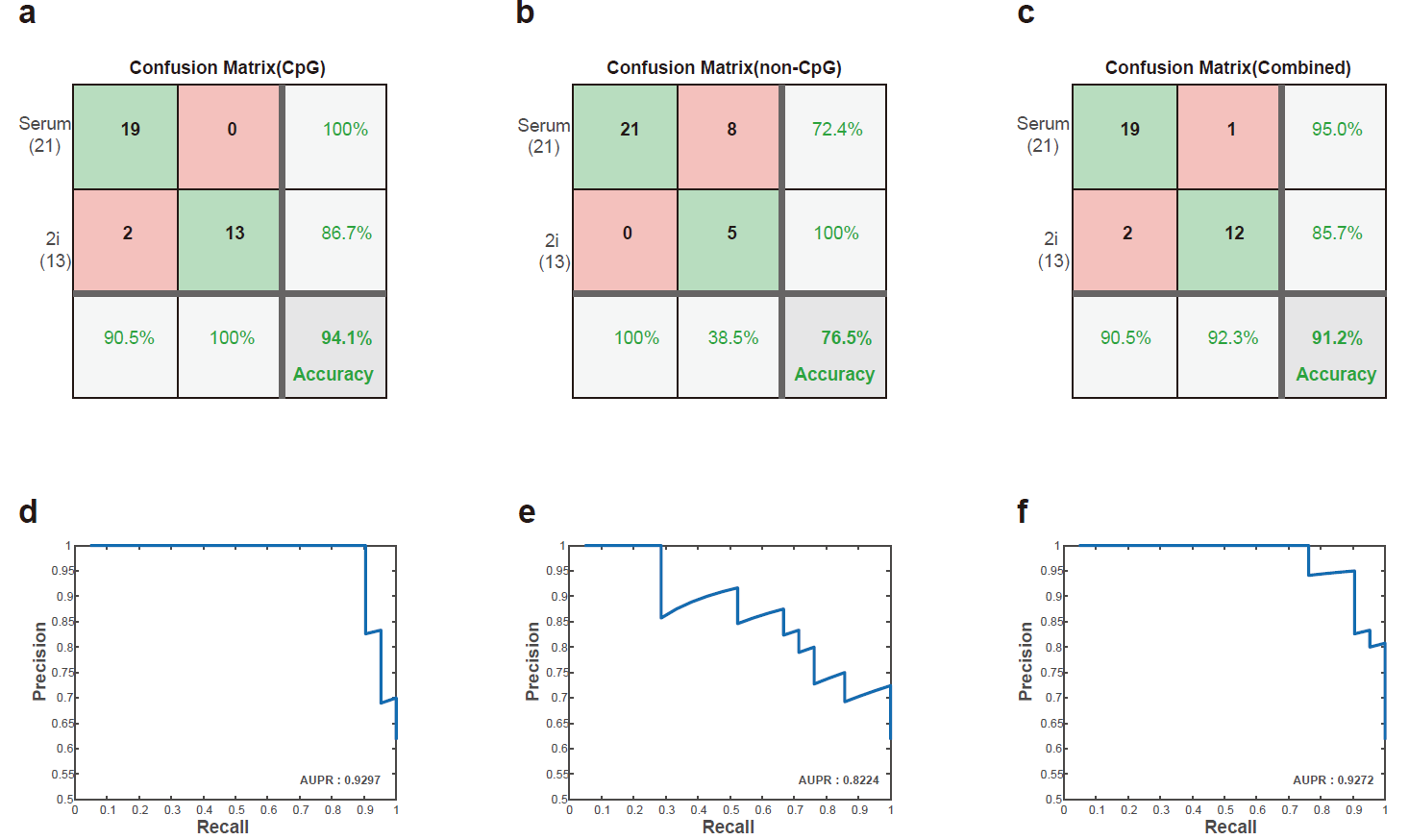


(a-c). Confusion matrices of three prediction models for GSE56879. On the confusion matrix plot, the rows correspond to the predicted serum and 2i groups, and the columns correspond to the true class. The diagonal cells correspond to cells that are correctly classified. The off-diagonal cells correspond to incorrectly classified cells. The column on the far right of the plot shows the percentages of all cells predicted to belong to each class that was correctly classified. The row at the bottom of the plot shows the percentages of all cells belonging to each class that was correctly classified. The cell in the bottom right of the plot shows the overall accuracy. (d-f). Precision-recall curves of three prediction models for GSE56879. Cells grown in serum environment are labeled true. AUPR represents area under precision recall curve.

### Supplementary Figure 5. Distributions of estimated cell pseudo-times by linear regression analysis.


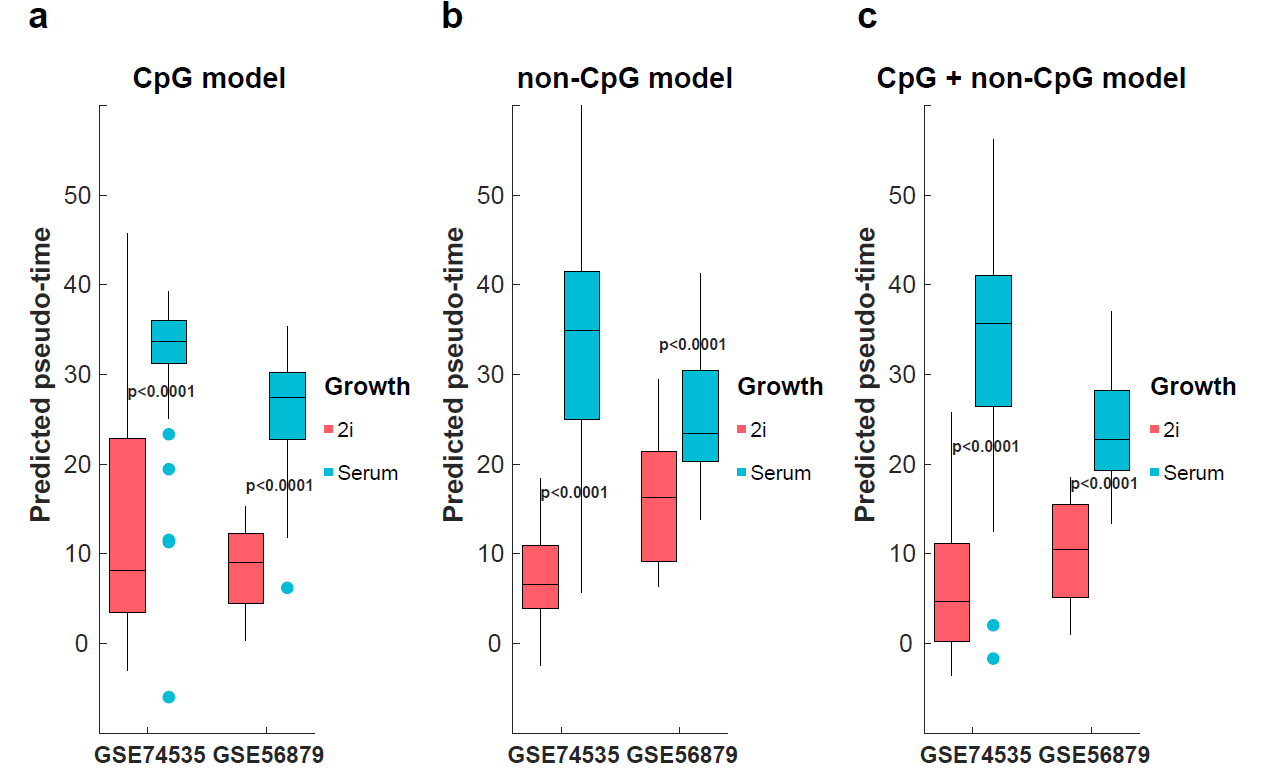


(a-c). Boxplots of cell pseudo-times according to cell culture environment in the CpG methylation model, non-CpG methylation model, and the combined model. Red boxes represent pseudo-times for cells grown in 2i media, and blue boxes represent cells cultured in serum. *p* represents the significance of differences in overall methylation levels between 2i and serum culture groups according to t-test.

### Supplementary Figure 6. A sliding window approach to define methylation levels at each genomic interval.


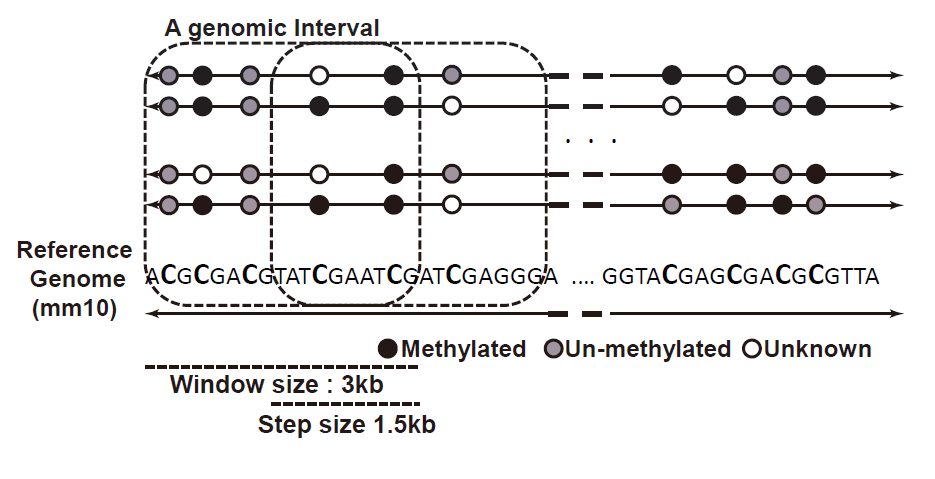


For the calculation of interval methylation levels, the window size was fixed at 3,000 bp, and the step size was fixed at 1,500 bp. We also included CHG and CHH (in which H = A, T or C) sites for non-CpG methylation, as well as CpG sites, when measuring DNA methylation levels in each interval. According to the type of each cytosine, the number of intervals comprised 420 at CpG and 3554 at non-CpG sites.

### Supplementary Figure 7. Selection of λ values of pluripotency prediction models


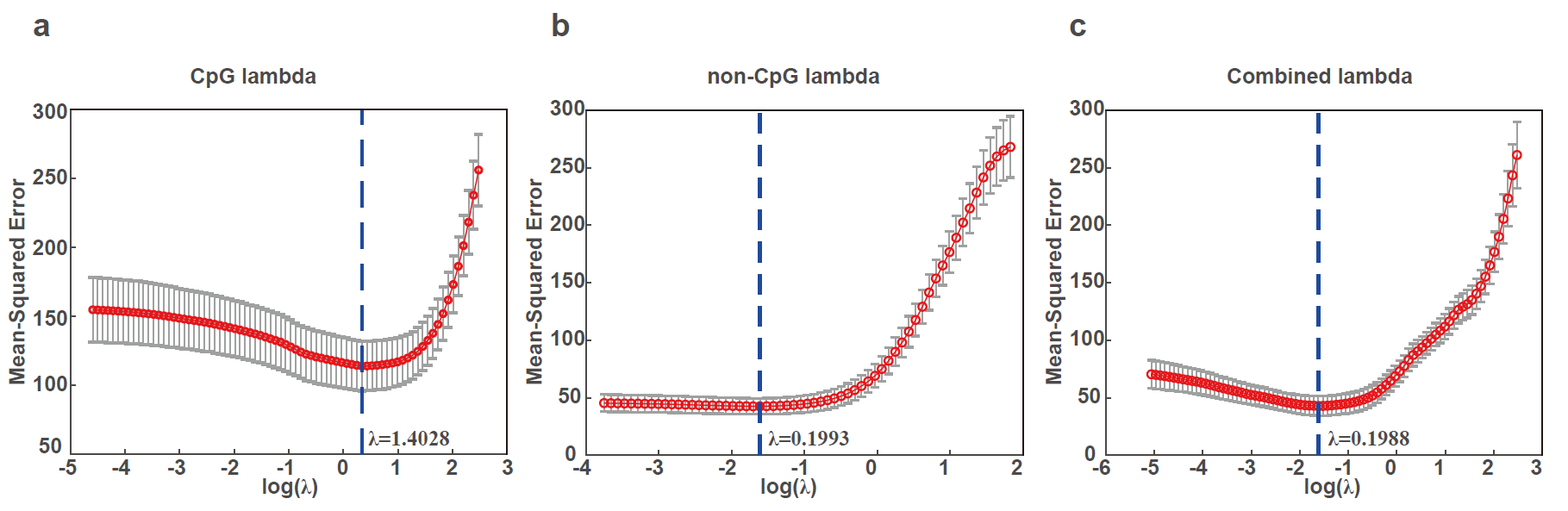


(a-c). 10-fold cross validation was performed using GSE74535 to select final parameters for each model. λ values were selected according to the minimum root mean square error.
