# Supplementary material for: Prediction model construction of mouse stem cell pluripotency using CpG and non-CpG DNA methylation markers
Source: BMC Bioinformatics. 2020 May 4;21:175. doi: 10.1186/s12859-020-3448-3 (PMC7199378; doi:10.1186/s12859-020-3448-3)
Supplement: Supplementary file 2 — Additional file 2: Table S1. List of the 16 CpG and 33 non-CpG genomic ranges used in the combined prediction model. Each of the chr, start, and end columns indicate chromosome and location information. R is the Pearson’s correlation coefficient, and p is the f-test result p-value. The type column indicates a CpG or non-CpG region. [file 12859_2020_3448_MOESM2_ESM.docx]

## Additional File 2. Supplementary Table

### Supplementary Table 1. List of the 16 CpG and 33 non-CpG genomic ranges used in the combined prediction model. In total, 49 methylation interval markers were used in the pluripotency prediction model. Each of the chr, start, and end columns indicate chromosome and location information. R is the Pearson's correlation coefficient, and p is the f-test result p-value. The type column indicates a CpG or non-CpG region.

| **Index** | **Chr** | **Start** | **End** | **R** | ***p-value*** | **Type** |
| --- | --- | --- | --- | --- | --- | --- |
| CG 1 | chr2 | 5494501 | 5497500 | 0.277384 | 0.015981 | CpG |
| CG 2 | chr1 | 188268001 | 188271000 | 0.391245 | 0.000519 | CpG |
| CG 3 | chr1 | 9544501 | 9547500 | 0.394014 | 0.00047 | CpG |
| CG 4 | chr15 | 92590501 | 92593500 | 0.42005 | 0.000176 | CpG |
| CG 5 | chr18 | 68217001 | 68220000 | 0.503052 | 4.23E-06 | CpG |
| CG 6 | chr4 | 4047001 | 4050000 | 0.5603 | 1.72E-07 | CpG |
| CG 7 | chr14 | 19417501 | 19420500 | 0.589845 | 2.57E-08 | CpG |
| CG 8 | chr5 | 144789001 | 144792000 | 0.636271 | 8.53E-10 | CpG |
| CG 9 | chr12 | 3109501 | 3112500 | 0.63673 | 8.23E-10 | CpG |
| CG 10 | chr1 | 85597501 | 85600500 | 0.672118 | 4.05E-11 | CpG |
| CG 11 | chr11 | 3169501 | 3172500 | 0.676194 | 2.79E-11 | CpG |
| CG 12 | chr1 | 85599001 | 85602000 | 0.702028 | 2.26E-12 | CpG |
| CG 13 | chr11 | 3177001 | 3180000 | 0.706916 | 1.36E-12 | CpG |
| CG 14 | chr2 | 168145501 | 168148500 | 0.711156 | 8.72E-13 | CpG |
| CG 15 | chr1 | 85338001 | 85341000 | 0.729635 | 1.13E-13 | CpG |
| CG 16 | chr17 | 3081001 | 3084000 | 0.749015 | 1.09E-14 | CpG |
| nonCG 1 | chr1 | 133891501 | 133894500 | -0.36084 | 0.001471 | Non-CpG |
| nonCG 2 | chr15 | 37243501 | 37246500 | -0.35322 | 0.001881 | Non-CpG |
| nonCG 3 | chr7 | 131590501 | 131593500 | -0.32049 | 0.005058 | Non-CpG |
| nonCG 4 | chr17 | 25477501 | 25480500 | -0.31879 | 0.005311 | Non-CpG |
| nonCG 5 | chr7 | 53497501 | 53500500 | -0.31685 | 0.005611 | Non-CpG |
| nonCG 6 | chr18 | 71598001 | 71601000 | -0.3163 | 0.005699 | Non-CpG |
| nonCG 7 | chr16 | 23799001 | 23802000 | -0.29807 | 0.009396 | Non-CpG |
| nonCG 8 | chr8 | 72703501 | 72706500 | -0.27549 | 0.016745 | Non-CpG |
| nonCG 9 | chr12 | 103644001 | 103647000 | -0.27253 | 0.018006 | Non-CpG |
| nonCG 10 | chr19 | 42991501 | 42994500 | -0.26028 | 0.024116 | Non-CpG |
| nonCG 11 | chr4 | 11718001 | 11721000 | -0.25933 | 0.024656 | Non-CpG |
| nonCG 12 | chr18 | 53329501 | 53332500 | -0.25386 | 0.027968 | Non-CpG |
| nonCG 13 | chr18 | 48496501 | 48499500 | -0.2473 | 0.032433 | Non-CpG |
| nonCG 14 | chr14 | 31137001 | 31140000 | -0.2465 | 0.033013 | Non-CpG |
| nonCG 15 | chr8 | 126948001 | 126951000 | -0.23838 | 0.039437 | Non-CpG |
| nonCG 16 | chr3 | 142002001 | 142005000 | 0.227614 | 0.049536 | Non-CpG |
| nonCG 17 | chr9 | 66799501 | 66802500 | 0.229547 | 0.047582 | Non-CpG |
| nonCG 18 | chr6 | 67006501 | 67009500 | 0.256827 | 0.02613 | Non-CpG |
| nonCG 19 | chr15 | 46867501 | 46870500 | 0.259413 | 0.02461 | Non-CpG |
| nonCG 20 | chr6 | 148492501 | 148495500 | 0.259894 | 0.024336 | Non-CpG |
| nonCG 21 | chr10 | 22353001 | 22356000 | 0.288217 | 0.012157 | Non-CpG |
| nonCG 22 | chr2 | 66555001 | 66558000 | 0.295262 | 0.01012 | Non-CpG |
| nonCG 23 | chr13 | 85713001 | 85716000 | 0.296904 | 0.00969 | Non-CpG |
| nonCG 24 | chr1 | 85602001 | 85605000 | 0.304032 | 0.008005 | Non-CpG |
| nonCG 25 | chr9 | 64303501 | 64306500 | 0.313575 | 0.006153 | Non-CpG |
| nonCG 26 | chr3 | 126373501 | 126376500 | 0.315452 | 0.005837 | Non-CpG |
| nonCG 27 | chr17 | 38680501 | 38683500 | 0.326615 | 0.004237 | Non-CpG |
| nonCG 28 | chr6 | 95214001 | 95217000 | 0.336557 | 0.003154 | Non-CpG |
| nonCG 29 | chr8 | 20394001 | 20397000 | 0.34699 | 0.002289 | Non-CpG |
| nonCG 30 | chr1 | 88272001 | 88275000 | 0.358609 | 0.001582 | Non-CpG |
| nonCG 31 | chr1 | 195369001 | 195372000 | 0.379803 | 0.000777 | Non-CpG |
| nonCG 32 | chr4 | 58032001 | 58035000 | 0.380626 | 0.000755 | Non-CpG |
| nonCG 33 | chr2 | 174102001 | 174105000 | 0.39147 | 0.000515 | Non-CpG |
